# THE NARRATIVES OF NURSING HOME RESIDENTS WITH PERSISTENT PAIN ABOUT THE PAINCQ-33

**DOI:** 10.1093/geroni/igad104.2588

**Published:** 2023-12-21

**Authors:** Timothy O’Connor

**Affiliations:** Nova Southeastern University, Davie, Florida, United States

## Abstract

**Problem:**

Persistent pain in older adults, often multimodal, remains poorly managed among nursing home residents. The resident’s perceptual barriers can interfere with how clinicians treat pain. Current pain instruments do not identify how older nursing home residents perceive interdisciplinary pain management care (IPMC). Purpose: This study explores the use of the Pain Care Quality (PainCQ-33) to measure how older nursing home residents with persistent pain perceive their IPMC and how these individuals communicate their persistent, multimodal pain to caregivers. Furthermore, this research determines how older residents prioritize their pain management expectations for maintaining their quality of life. Research design: A focus group design was used to explore residents’ viewpoints about the ability of the PainCQ-33 to accurately measure the perception of the quality of their pain management care. Data analysis: Transcripts and field notes from focus group discussions were analyzed using descriptive content analysis.

**Results:**

The identified codes illustrate how pain interference affects the ratings an older resident assigns to their pain management care and expectations of whether their pain can be relieved. Additional codes demonstrate an older resident’s pain-relieving measures and who they identify as helping to alleviate their pain. A theme from the codes emerged, where the relevance of pain management was based on the caring nature for chronic pain, which is the underpinning of the PainCQ-33 instrument. Significance: Clinicians, including nurses, can benefit from having a pain assessment tool uniquely designed to assess how this population perceives the quality of their pain management care.

